# Evaluation of the effects of exogenous cortisol manipulation and the glucocorticoid antagonist, RU486, on the exploratory tendency of bluegill sunfish (*Lepomis macrochirus*)

**DOI:** 10.1007/s10695-023-01250-0

**Published:** 2023-10-11

**Authors:** Annaïs Carbajal, Michael J. Lawrence, Kathleen M. Gilmour, Manel Lopez-Bejar, Steven J. Cooke

**Affiliations:** 1 Department of Animal Health and Anatomy, Veterinary Faculty, UniversitatAutònoma de Barcelona, 08193 Bellaterra, Barcelona, Spain; 2 Fish Ecology and Conservation Physiology Laboratory, Department of Biology, Carleton University, Ottawa, ON Canada; 3 Department of Biological Sciences, University of Manitoba, Winnipeg, MB Canada; 4 Department of Biology, University of Ottawa, Ottawa, ON Canada; 5 College of Veterinary Medicine, Western University of Health Sciences, Pomona, CA USA

**Keywords:** Teleost, Stress, Behaviour, Endocrinology, Glucocorticoid receptor, Boldness

## Abstract

In teleost fishes, activation of the hypothalamic-pituitary-interrenal axis leads to an elevation of circulating cortisol levels as a primary stress response. While acute elevation of cortisol is generally beneficial, long-term elevation, a common characteristic of chronic stress, may lead to detrimental effects on health and physiological performance in fishes. Some stress-mediated behavioural shifts, such as variation along the shy-boldness axis in fish, may influence individual fitness. The present study evaluated the role of cortisol and its mechanisms of action in the exploratory behaviour of the bluegill sunfish (*Lepomis macrochirus*). Fish were implanted with cocoa butter alone (sham treatment), or cocoa butter containing cortisol, or cortisol and the glucocorticoid receptor antagonist, RU486. A control (untreated) group was also used. Animals were held for 48 h following treatment and then were subjected to a Z-maze trial to characterize the exploratory behaviour. Cortisol treatment had no measurable effect on the exploratory behaviour of bluegill sunfish. Despite presenting a higher probability of refuge emergence, fish treated with cortisol combined with RU486 behaved similarly to cortisol-treated and control groups. While these results suggest that cortisol may not be involved in the mechanisms controlling boldness, the influence of cortisol elevation across longer time periods plus validation in different contexts will be necessary to confirm this conclusion.

## Introduction 

Glucocorticoids (GC), the end-product of the hypothalamic-pituitary-interrenal (HPI) axis, are a main component of the physiological stress response in teleost fishes (Moberg and Mench 2000; Wendelaar Bonga 1997; Schreck and Tort 2016) as in other vertebrates (Moberg and Mench 2000). Cortisol, the principal GC in teleost fishes, stimulates physiological processes such as gluconeogenesis to mobilize energy reserves at the expense of other functions such as growth, reproduction, or immune function (Sapolsky 2002; Schreck & Tort 2016). In responses to an acute stressor, short-term HPI axis activation is associated with a suite of adaptive behavioural responses that favour survival, such as increased foraging behaviour and promotion of escape (Pankhurst 2011; Ellis et al. 2012). In this manner, the stress response enables the organism to cope with a potentially harmful threat (Barton 2002). However, long-term HPI axis activation (i.e., via chronic stress) can have detrimental effects on health and physiological performance (Branson 2008; Schreck & Tort 2016). Stressors can lead to reduced growth and reproduction, compromise the immune system, and impair the animal’s ability to cope with environmental challenges (Wendelaar Bonga 1997; Le Moal 2007; Fuzzen et al. 2011; Sadoul and Vijayan 2016).

Despite the importance of the stress response in allowing animals to respond to diverse and often unpredictable challenges (Angelier & Wingfield 2012), the mechanisms through which cortisol modifies behavioural patterns remain poorly understood in wild teleost fishes. Cortisol may exert effects via two pathways, a fast non-genomic route, activated by membrane-mediated signalling cascades, and a slower genomic route which involves the transfer of cortisol across the cell membrane to regulate target gene transcription and translation (Moore and Evans 1999; Sørensen et al. 2013; Das et al. 2018). To date, two intracellular receptor types have been identified in teleost fishes, the glucocorticoid receptor (GR), of which there are two paralogs in most teleost fishes (GR1 and GR2), and the mineralocorticoid receptor (MR) (Bury et al. 2003; Stolte et al. 2006). Mineralocorticoid receptors have a higher affinity for cortisol and are usually activated at lower cortisol concentrations than GRs (Bury & Sturm 2007). Evidence for actions of cortisol mediated through GR binding arises from studies using the receptor antagonist, RU486 (Roy and Rai 2009; Espinoza et al. 2017). Most published studies have focused on the involvement of GR in modulating fish physiology (Vijayan and Leatherland 1992; Shaw et al. 2007; Teles et al. 2013), while the involvement of these receptors in modulating fish behaviour is still unclear (Best and Vijayan 2018; Lawrence et al. 2019b; Ros et al. 2012). 

From an ecological perspective, the influence of stress on fish behaviour is relevant to understanding evolutionary trade-offs in nature. Boldness, defined as the tendency of an animal to take risks and be exploratory in novel environments (Lima & Dill 1990), influences individual fitness and plays an important role in evolutionary processes (Wilson & Godin 2009). Many efforts have been made to find out how fish adjust this behaviour in different environmental contexts. Although many behaviours are in some measure heritable (Dingemanse et al. 2012; Ariyomo et al. 2013), they can be shaped by environmental challenges and as such, animals can modify their boldness phenotype in relation to the stress experienced (Godin and Smith 1988; Killen et al. 2013). A common practice to evaluate how stress influences fish behaviour is to administer exogenous GC; however, this approach has to date produced diverse results (Sopinka et al. 2015; Crossin et al. 2016). Previous work has shown that exogenous cortisol implants can increase (O’Connor et al. 2010), decrease (Øverli et al. 2002; Algera et al. 2017a) or have no impact (Lawrence et al. 2018b, 2019a) on locomotor activity of fish, highlighting the complexity of the interaction between stress and fish behaviour. Research focused on the connection between behaviour and energetics without GC manipulation showed that fish experiencing different metabolic challenges, such as hunger or parasitism, tended to behave in a more risky manner, spending less time beneath a refuge or increasing their activity levels (Godin and Smith 1988; Killen et al. 2013). This finding suggests that variation along the boldness axis could be mediated in part by fluctuations of cortisol as a function of energy metabolism. However, divergent findings emphasize the need to further investigate the relationship between the HPI axis and fish behaviour. The currently described effects of cortisol on physiology are mediated predominantly through activation of GRs (Mommsen et al. 1999; Shaw et al. 2007; Teles et al. 2013), but the contribution of this receptor to modulating fish behaviour needs to be further studied.

Therefore, the purpose of the present study was to test the hypothesis that chronic cortisol elevation would alter exploratory behaviour. We predicted that bluegill sunfish (*Lepomis macrochirus*) with elevated cortisol levels would exhibit increased exploratory behaviour compared to sham-treated (implanted with cocoa butter alone) and control (untreated) individuals. Fish treated with RU486, which was expected to block cortisol-mediated effects on fish behaviour, were predicted to exhibit an exploratory tendency comparable to that observed in control and sham individuals. We used bluegill sunfish as a common model in behavioural ecology and animal physiology that is also ecologically relevant as dominant fish community members in many northern temperate lakes in North America. This work contributes to the growing body of studies that consider the manifold role of corticosteroids (beyond just a stress hormone) in wild animals (see MacDougall-Shackleton et al. 2019).

## Materials and methods

### Cortisol time course and dosage validation

To select the dose and time course for cortisol treatment, a group of 120 bluegill sunfish was captured by seine net along the shore of Lake Opinicon (Ontario, Canada) on May 27, 2017 (water temperature ~ 19 °C). Fish were transported back to the Queen's University Biological Station (QUBS; Elgin, Ontario, Canada) immediately after being caught. Experimental series followed the guidelines of the Canadian Council on Animal Care under administration of the Carleton University Animal Care Committee (AUP 104281). Animals were randomly assigned to one of three treatment groups: implanted with cocoa butter alone (sham treatment; 5 ml cocoa butter/kg BW), implanted with cocoa butter containing a low-dose cortisol (25 mg cortisol/kg BW) and implanted with cocoa butter containing a high-dose cortisol (50 mg cortisol/kg BW). Implants consisted of cocoa butter that was warmed until it was liquid and injected using a 1 ml syringe and an 18G needle into the coelom of the fish just posterior to the pelvic fins (Gamperl et al., 2019; Lawrence et al. 2018a,b; 2019a, b). Cocoa butter was used for cortisol administration since this vehicle has proven efficient for rapid and sustained elevation of plasma cortisol for several days (Gamperl et al. 1994; Sopinka et al. 2015) including for bluegill (McConnachie et al. 2012b). The dosage validation did not contain a no-injection control group nor a group implanted with cortisol combined with RU486 because we aimed to identify the cortisol dosage that would produce a physiologically-relevant elevation of cortisol levels. Moreover, we decided not to include the non-treated control group because in bluegill the implant administration is unlikely to influence stress parameters beyond a brief acute stress response from handling (McConnachie et al. 2012b). To ensure even distribution of cortisol throughout the cocoa butter, the procedure of Hoogenboom et al., (2011) was employed. Briefly, crystalline cortisol (hydrocortisone 21-hemisuccinate; Sigma H4881, Sigma-Aldrich, Oakville, Ontario, Canada) was dissolved in ethanol and mixed with melted cocoa butter. The ethanol was then evaporated, and 0.5 ml of the mixture was drawn into a 1 ml syringe. Once the mixture reached ambient temperature it was stored at − 20◦ C until its use at which time it was again melted for injection. The use of coelomic implants for hormone delivery is considered a standard procedure in teleost fishes (Gamperl et al. 1994) that has been used extensively in centrarchid fishes (e.g. Algera et al. 2017a; Lawrence et al. 2019b; McConnachie et al. 2012b; Rodgers et al., 2012; Zolderdo et al., 2016). Following administration of the implant, fish were maintained in the holding tanks (~ 212 L) as a single shoal per treatment group. Blood samples were collected at 12, 24, 48 or 96 h after implantation. Previous research on bluegill has used similar timepoints for validation studies and found that this sampling strategy enables one to fully assess exogenously-manipulated cortisol dynamics for this species (McConnachie et al. 2012b). At each time point, 10 individuals of each treatment group were sampled. Blood was withdrawn by caudal puncture using a heparinized (Na^+^ heparin, 10, 000 USP units/ml; Sandoz Canada Inc., Boucherville, QC, Canada) 1 ml syringe and 23 G needle (Lawrence et al. 2020). To avoid cortisol elevation induced by the stress of capture, blood collection occurred within 3 min of netting the fish (Lawrence et al. 2018c). Whole blood [glucose] was analysed immediately using a portable point-of-care device (Accu Chek Compact Plus; Hoffman, La Roche Limited, Mississauga, ON, Canada) previously validated for use in teleost fish (Beecham et al. 2006; Cooke et al. 2008; reviewed in Stoot et al., 2014). The remaining blood was centrifuged at 2000 g (Mandel Scientific, Guelph, ON, Canada) for 2 min and plasma was collected and stored at -80 ºC for later analysis of plasma cortisol titres. Plasma cortisol levels were analysed using a commercial radioimmunoassay kit (ImmuChem Cortisol Coated Tube RIA Kit, MP Biomedicals, Solon, OH, USA) previously validated for use in teleost fishes (Gamperl et al. 1994). All samples were analysed in a single assay where intra-assay variability (% CV) was 7.13%. Fish were euthanized by cerebral percussion and total length, body weight, and liver mass were recorded. Liver mass was used to calculate the hepatosomatic index [HSI = (liver mass/body mass) × 100%; Busacker et al. 1990], an index of individual energetic state that has been previously applied as a stress indicator (Sopinka et al. 2016).

### Experimental animals

For the behavioural trials, a separate group of bluegill sunfish (*Lepomis macrochirus* Rafinesque, 1819; total length = 9.8 ± 0.6 cm; mass = 13.9 ± 2.4 g; mean ± SD, *N* = 100) was captured as mentioned previously, over a 4-day period (*N* = 25 fish captured per day) in mid-June 2017 (after the spring reproductive period for bluegill) from Lake Opinicon. Fish were quickly transported back to the Queen's University Biological Station. Bluegill were allowed to acclimate and recover for a 24 h period after being caught. The acclimation period was based on previous studies in bluegill by some of our team (e.g., Lawrence 2019; Moynes et al. 2020) as well as observations by other researchers that this species acclimatizes easily to laboratory conditions (Wilson & Godin 2009). Bluegill were kept in indoor flow-through tanks (~ 212 L) supplied with flowing lake water at ambient temperature (23.4 ± 0.8 ºC) under natural photoperiod. Hunger state can potentially influence boldness behaviours (e.g., Gotceitas and Godin 1991; Godin and Smith 1988; Killen et al. 2011). As such, fish were not fed while kept in captivity to prevent any potential confounding effects. Previous research on bluegill has revealed that withholding of food for several days does not yield biologically meaningful reductions in fish condition (Lawrence et al. 2019c) presumably given the transient nature of food resources in nature.

Experimental animals were randomly selected from the holding tanks and assigned to one of the four treatments: control (no implant), implanted with cocoa butter alone (sham treatment), implanted with cocoa butter containing cortisol (cortisol treatment), or implanted with cocoa butter containing cortisol and the GR antagonist, RU486 (cortisol + RU486 treatment). To avoid temporal bias, the order of implantation with each treatment was altered daily and was conducted in a systematically random fashion. Cocoa butter alone (i.e. sham implant; 5 ml/kg body weight [BW]), or cocoa butter containing cortisol (hydrocortisone 21-hemisuccinate; 25 mg/kg BW), or cortisol combined with RU486 (25 mg cortisol/kg BW + 100 mg RU486/kg BW; Doyon et al. 2006; Lawrence et al. 2017) was injected as described previously; the cortisol dose was chosen on the basis of pilot experiments (see above). We also included a no-injection control group that was otherwise handled identically to treated fish to account for stress and other factors associated with the procedure itself (McConnachie et al. 2012a). Immediately after administering the implant, dorsal fin spines were clipped (with unique patterns) to identify treatment groups. Fish were then transferred to a holding tank (~ 406 L) under the conditions described above for 48 h until behavioural assays were performed. This holding period was used to allow the implanted cortisol to reach ecologically relevant circulating concentrations and ensure that levels of the hormone were elevated at a level that was reflective of a stressed state. The same experimental tank was used for all treatment groups to eliminate variability due to the potential tank effect. 

### Behavioural analysis

**Fig. 1:**
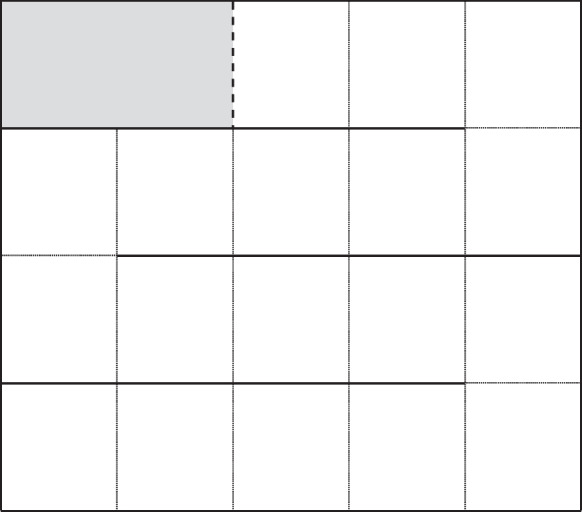
Representation of the Z-maze with the refuge box (grey rectangle) and 18 zones delimited by dotted lines. After one minute of acclimation, the gate of the refuge was remotely opened (dashed lines), and each individual had 5 min to explore the maze

Behavioural assessments of control (*n* = 19), sham (*n* = 18), cortisol-treated (*n* = 18), and cortisol-plus RU486-treated (*n* = 21) fish were performed 48 h after implant injection. The treatment exposure period of 48 h was selected on the basis of pilot experiments (see above). Since fish were captured over a 4-day period, behavioural tests were carried out during four consecutive days. Every day, a group of 25 fish from all four experimental groups (control, sham, cortisol and cortisol plus RU486 treated 48 h before) were behaviourally tested. The 4-day experimental period allowed the daily assessment of a reduced group of fish in a short time frame and thus avoid the circadian rhythm be a source of variability in cortisol levels (Dickmeis 2008). One Z-maze, placed next to the holding tanks, was used to evaluate exploratory activity following methods described in previous studies (Chapman et al. 2010; Lawrence et al. 2018b). The maze (40 cm × 50 cm) was configured with 4 corridors, each divided into equal quarters to yield a total of 18 zones and had a refuge in one corner (Fig. 1). During trials, the maze was illuminated with artificial light from above the tank, and the behaviour of the test fish was recorded using a single Go-pro Hero camera (Go-Pro, San Mateo, CA, USA) situated above the maze. The maze was filled to a depth of 6.6 cm with fresh lake water that was changed after each trial.

At the onset of a trial, a fish was placed into the refuge zone with the help of a hand net and a small bucket and was allowed one minute of acclimation (Chapman et al. 2010; Wood et al. 2011; Raoult et al. 2012). The experiment began when the door between the refuge and the maze was opened remotely by an observer behind a blind. Fish were given 10 min to leave the refuge, and if the individual did not leave the refuge during this time, the trial was ended. If the fish did emerge, the animal was given 5 additional minutes to explore the refuge. During the trial, we recorded the latency to emerge from the refuge once the door had been opened, the number of lines crossed (i.e. activity levels), and the time to reach the end of the maze for the first time. Additionally, if the individual returned to the refuge after emerging, the total time spent in the refuge during the 5 min trial also was recorded. The proportion of fish that did not exit the refuge, and the proportion that did not reach the end of the maze also were recorded. At the end of the trial, fish were euthanized via cerebral percussion and body weight and total length were measured.

### Statistical analysis

Statistical analyses were conducted using R software (R-project, Version 3.5.1, R Development Core Team, University of Auckland, New Zealand), and the level of significance for all tests was 0.05. Data were tested for normality using the Shapiro–Wilks test and for homogeneity of variance with Levene’s test. Data were square-root or log transformed to meet assumptions of normality and equal variance where appropriate.

For the dose and time-course validation, two-way analysis of variance (ANOVA) followed by post-hoc Tukey’s HSD test, as appropriate, was used to test whether cortisol and blood glucose concentrations, and HSI, differed significantly across treatment groups (sham, low-dose and high-dose cortisol) over the sampling times (12, 24, 48 and 96 h).

Chi-square tests were used to assess the significance of associations between treatment group (control, sham, cortisol alone, and cortisol combined with RU468) and 1) the probability of exiting the refuge, and 2) the probability of reaching the end of the maze. Because all individuals treated with cortisol plus RU468 exited the refuge, a second chi-square test was performed to test for differences among control, sham and cortisol treatments. To determine whether there were significant differences among treatment groups in 1) the latency to emerge from the refuge, 2) the number of lines crossed, 3) the time to reach the end of the maze, and 4) the total time spent in the refuge during the 5 min trial, general linear models (GLM) were used. Water temperature and fish total length were added to the models as linear covariates, as was the interaction of these covariates with treatment group.

## Results

### Time course and cortisol dose validation

**Fig. 2:**
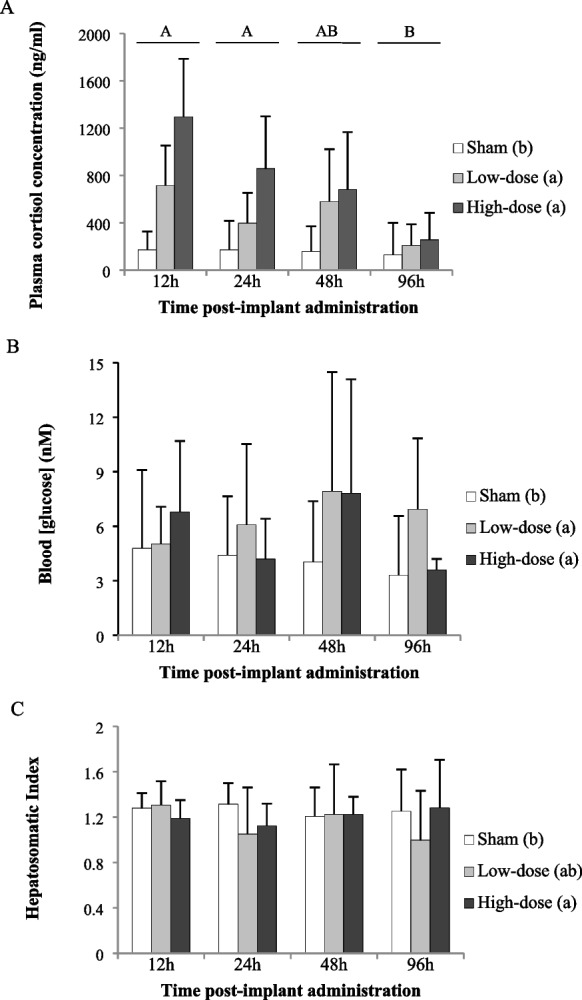
Time course elevation and dosage validation for bluegill sunfish (Lepomis macrochirus). Plasma cortisol concentrations (**A**), blood glucose concentrations (**B**) and hepatosomatic index (**C**) of sham (implanted with cocoa butter alone), low-dose cortisol (25 mg/kg BW) and high-dose cortisol (50 mg cortisol/kg BW) treatments sampled from 12 to 96 h after implant administration. Values are reported as means ± standard deviation (SD; error bars). Capital letters represent significant effects of time (*p* < 0.001) and lower case letters represent significant effects of dose (*p* < 0.05). The interaction of these two factors was not significant (*p* = 0.95). At every dose (sham, low-dose and high-dose cortisol) and time period (12 h, 24 h, 48 h and 96 h), the sample size was 10 individuals

Treatment group (F = 35.155; df = 2; *p* < 0.001) and sampling time (F = 6.645; df = 3; *p* < 0.001) significantly affected circulating cortisol concentrations (Fig. 2A), but the interaction of these factors was not significant (F = 0.264; df = 6; *p* = 0.95). Individuals from the low- and high-dose cortisol treatments presented higher cortisol values than sham fish (Tukey HSD, *p* < 0.001). At 96 h, cortisol values were lower than those of fish sampled at 12 h (Tukey HSD, *p* < 0.001) and at 24 h (Tukey HSD, *p* = 0.03). Blood glucose and HSI were influenced by treatment (glucose, F = 7.207, df = 2, *p* < 0.001, Fig. 2B; HSI, F = 3.898, df = 2, *p* = 0.02, Fig. 2C) but not by sampling time (glucose, F = 1.054, df = 3, *p* = 0.37; HSI, F = 0.592, df = 3, *p* = 0.64). The interaction of these factors was not significant (glucose, F = 1.094, df = 6, *p* = 0.37; HSI, F = 1.891, df = 6, *p* = 0.09). Sham individuals differed from fish in both the low- (Tukey HSD, *p* = 0.001) and high-dose (Tukey HSD, *p* = 0.03) cortisol treatments in blood glucose levels. In HSI, differences were close to significant between sham and low-dose cortisol (Tukey HSD, *p* = 0.06) and significant between sham and high-dose cortisol treatment groups (Tukey HSD, *p* = 0.04).

### Behavioural responses

A Chi-square test revealed that treatment had a significant effect on the probability to exit the refuge when all the treatments were considered in the analysis (χ^2^ = 9.043, df = 3, *p* = 0.03; Table 1). After excluding the cortisol treatment combined with RU486 from the test, the chi-square analysis revealed no significant differences among control, sham and cortisol-treated groups (χ^2^ = 2.400, df = 3, *p* = 0.30). No significant differences were detected among groups for the probability of reaching the end of the maze (χ^2^ = 4.5623, df = 3, *p* = 0.21; Table 1).

**Table 1:** Distribution of treatment groups (control—not implanted, sham—implanted with cocoa butter alone, cortisol alone—25 mg/kg BW and cortisol combined with RU486—25 mg cortisol/kg BW + 100 mg RU486/kg BW) between the number of individuals (%) that did (Yes) or not (No) exit the refuge box and the number of individuals (%) that reached (Yes) or not (No) the end of the maze

		Treatment			
		Control	Sham	Cortisol	Cortisol + RU486
Exit	Yes	13 (68%)	13 (72%)	16 (89%)	21 (100%)
	No	6 (32%)	5 (28%)	2 (11%)	0 (0%)
End	Yes	7 (37%)	8 (44%)	11 (61%)	14 (67%)
	No	12 (63%)	10 (56%)	7 (39%)	7 (33%)

**Fig. 3:**
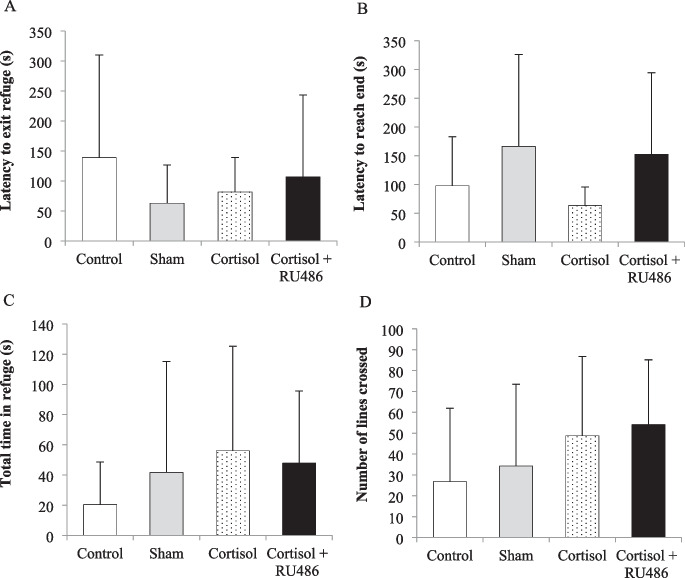
Metrics for control—not implanted (white bars; *n* = 19), sham—implanted with cocoa butter alone (grey bars; *n* = 18), cortisol alone—25 mg/kg BW (dotted bars; *n* = 18) and cortisol combined with RU486—25 mg cortisol/kg BW + 100 mg RU486/kg BW (black bars; *n* = 21) bluegill sunfish in the maze trial displaying (**A**) the latency to exit the refuge, (**B**) the latency to reach the end of the maze, (**C**) the total time spent in the refuge box, and (**D**) the number of lines crossed. Values are shown as mean and error bars are standard deviations

No differences were detected among treatments in the latency to exit the refuge (F = 1.070; df = 3; *p* = 0.37; Fig. 3A), the latency to reach the end of the maze (F = 1.751; df = 3; *p* = 0.18; Fig. 3B), the total time spent inside the refuge during the 5-min trial (F = 0.982; df = 3; *p* = 0.40; Fig. 3C), or the number of lines crossed (F = 2.512; df = 3; *p* = 0.07; Fig. 3D).

## Discussion

### Overview

Because of the influence of behaviour in evolutionary processes and individual fitness (Wilson & Godin 2009; Noakes and Jones 2016), we aimed to gain insight into the effects of the HPI axis in mediating fish behaviour by testing whether exploratory tendency in the bluegill sunfish is influenced by cortisol. Contrary to our predictions, cortisol treatment had no measurable effect on exploratory behaviour. Despite a higher probability of emergence from the refuge, fish treated with cortisol combined with RU486 behaved similarly to those in all other treatment groups.

### Validation of cortisol implants

The time-course of cortisol elevation achieved using the implants was monitored in a separate group of bluegill. Significant differences in circulating cortisol levels between fish treated with low and high cortisol dosages were not detected in the validation experiment. Nevertheless, subjects from the behavioural assessment were implanted with the low dose of cortisol (25 mg cortisol/kg BW). This implant was chosen to elevate plasma cortisol levels given that previous experiments have revealed that the use of high (supraphysiological) doses of cortisol can lead to altered behavioural responses which may not be of physiological relevance (McConnachie et al. 2012b; Lawrence et al. 2018a). Importantly, 48 h after the administration of a cocoa butter implant containing the low dose of cortisol (25 mg cortisol/kg BW), circulating hormone levels were similar to those detected in bluegill after either repeated daily stressors (Cook et al. 2012) or a severe acute stress (McConnachie et al. 2012b). Cortisol levels in cortisol-treated fish remained significantly higher than those of the sham group up to 48 h post implant administration, and, consistent with the gluconeogenic activity of this hormone (reviewed in Mommsen et al. 1999), cortisol-treated fish displayed elevated blood glucose after administration of the implant. Hence, the results of the time-course experiment suggest that the hormone dose used in the Z-maze trial under laboratory conditions would yield circulating cortisol levels that were physiologically relevant.

### Influence of cortisol on exploratory tendency

Cortisol elevation was predicted to increase the exploratory tendency of wild bluegill sunfish because considerable evidence suggests that fish experiencing energetic and metabolic challenges alter the degree of boldness towards risk-taking behaviours (Krause et al. 2000; Killen et al. 2011, 2013). Contrary to our predictions, we found that fish treated with cortisol for 48 h exhibited a behavioural phenotype comparable to that observed in control fish. Fish species differ considerably in their ability to cope with stressors through behavioural and/or physiological responses (Sopinka et al. 2015; Schreck and Tort 2016). For example, increased locomotor activity was observed in zebrafish larvae treated with cortisol for 24 h (Best & Vijayan 2018), and rainbow trout exhibited increased activity 1.5 h after being fed cortisol-treated food, which decreased by 48.5 h (Øverli et al. 2002). In zebrafish stressed without further GC manipulation, an effect on the activity was revealed as long as 14 d after the onset of the stressor (Piato et al. 2011; Manuel et al. 2014). An influence of cortisol may not be apparent if the time at which a behavioural assay is performed is too close to or too long after cortisol administration (Algera et al. 2017a, b; Zolderdo et al., 2016; Lawrence et al. 2019b). Although in the current study cortisol levels in bluegill sunfish remained elevated for 48 h, it is possible that this duration of cortisol exposure was too short to detect effects of cortisol on the behaviour of this species**.** In some cases, while elevated cortisol promotes specific physiological changes, behaviour may remain unaltered as an adaptive mechanism to avoid potential fitness consequences (Dunlap et al. 2011; Lawrence et al. 2019b; Piato et al. 2011). Consistent with our findings, negative results have been published in previous studies. For instance, no change in exploratory tendency was detected in cortisol-treated pumpkinseed sunfish in a study carried out under comparable experimental conditions (Lawrence et al. 2018b). Likewise, no effect of cortisol treatment on activity was observed in creek chub or pumpkinseed sunfish implanted with cortisol (Lawrence et al. 2019a; Nagrodski et al. 2013). While these findings may indicate that cortisol is not involved in exploratory behaviour, the complex relations between GC and other signalling systems suggest that this interpretation should be regarded with caution. For example, when faced with a secondary stressor (i.e. intruder test), cortisol-treated rainbow trout showed impaired locomotor performance, but this effect may not be detected in undisturbed conspecifics also treated with cortisol (Øverli et al. 2002). This scenario has long been observed in vertebrates with behaviours other than activity and locomotion (Crossin et al. 2016; Sopinka et al. 2015). Accordingly, context may play an important role in determining how GCs influence exploratory tendency, and a behavioural impairment may not be observed in all situations. We cannot rule out potential cortisol increases related to relocation from the holding tank to the Z-maze, which could have masked treatment differences. Unfortunately, circulating cortisol levels were not assessed in the experimental fish once the behavioural trial was finished. To further understand the role of cortisol in the exploratory tendency of bluegill fish it will be of interest to evaluate cortisol concentrations in the experimental individuals.

### Influence of RU486 on exploratory tendency

Fish implanted with cortisol plus RU486 behaved similarly to cortisol-treated and control groups once in the maze. However, the antagonist-treated individuals were more likely to emerge from the refuge into a novel environment than fish from other treatments. The GR antagonist RU486 blocks the cortisol-mediated negative feedback pathway that inhibits HPI axis activity (Bury et al. 2003; Roy and Rai 2009). As a consequence of GC feedback inhibition, higher plasma cortisol levels often are detected in RU486-treated fish compared to conspecifics treated with cortisol alone (Reddy et al. 1995; Veillette et al. 2007). Cumulative increases in cortisol exposure could lead to the higher proportion of RU486-treated individuals that exit the refuge. Existing literature supports the effectiveness of RU486 in inhibiting cortisol-mediated effects by GR signalling on fish behavioural and physiological traits (DiBattista et al. 2005; Schjolden et al. 2009; Dunlap et al. 2011). The behavioural effect observed in the present study therefore could be modulated via the MR, a pathway reported to have an important role in mediating effects of cortisol on behaviours in teleosts, particularly, in relation to locomotor activity (Stolte et al. 2008; Takahashi and Sakamoto 2013; Sakamoto et al. 2016; Faught and Vijayan 2018). Nevertheless, given that only one measurable effect was detected in the RU486-treated group among the several metrics evaluated, the biological relevance of this outcome requires careful consideration. Further studies are warranted to reveal whether MR are involved in the modulation of exploratory tendency.

## Conclusion

In conclusion, although cortisol implants elevated plasma cortisol levels, the experiments used in the present study did not provide evidence that cortisol shapes exploratory behaviour in bluegill sunfish. Behavioural assessments performed at different time points after cortisol exposure (> 48 h) will be necessary to further characterize the role of cortisol in regulating fish behaviour. Treatment with cortisol plus RU486 showed that antagonist treatment increased the probability of emergence from the refuge, although no differences were detected for the other metrics evaluated. We speculate that signalling through MR could be involved, but this possibility requires experimental support. In the present study, cortisol levels were not assessed in individuals used for behavioural trials, limiting the interpretation of our results. As with all studies involving experimental manipulation of GCs, it is important to note that cortisol treatment is not the same as cortisol elevation during a stress response in that the neurosensory cascade is bypassed (Sopinka et al. 2015). As such, there is need for additional research that considers the effects of diverse stressors on fish to investigate the context-specificity of GC effects on behaviour.

## Data Availability

The datasets generated during and/or analysed during the current study are available from the corresponding author on reasonable request.
